# Effect of Reverse Engineering Pedagogy on Primary School Students’ Computational Thinking Skills in STEM Learning Activities

**DOI:** 10.3390/jintelligence11020036

**Published:** 2023-02-15

**Authors:** Xiaohong Liu, Xiao Wang, Kexue Xu, Xiaoyong Hu

**Affiliations:** 1 School of Education Science, Nanjing Normal University, Nanjing 210097, China; 2 School of Information Technology in Education, South China Normal University, Guangzhou 510631, China; 3 Institute of Artificial Intelligence in Education, South China Normal University, Guangzhou 510631, China

**Keywords:** computational thinking skills, reverse engineering pedagogy, STEM learning activity, primary school student, a quasi-experimental study

## Abstract

Computational thinking (CT) is important for students because it is one of the 21st century’s skills. Reverse engineering pedagogy (REP) can improve students’ CT due to its ability to develop students’ cooperativity, algorithmic thinking, creativity, and problem-solving in discipline education. Thus, this study aimed to explore the effect of REP on primary school students’ CT skills in STEM learning activities. A total of 101 fifth graders in a primary school participated in the study for one semester (16 weeks), including 51 students in the experimental group (EG) with REP, and 50 students in the control group (CG) with the demonstration method (DM). The computational thinking scale (CTS) was used to measure the CT skills of students in the pretest and posttest. The Wilcoxon signed-rank test and the Mann-Whitney *U* test were used to analyze the data. The results verified that REP had a fine effect on the improvement of students’ CT skills compared to the DM. The findings can provide some ideas for researchers to develop students’ CT skills in STEM learning activities. Teachers can use different teaching methods to reasonably arrange teaching activities to develop primary school students’ CT skills.

## 1. Introduction

Computational thinking (CT) is the thinking process which can formulate problems and their solutions (Wing 2011). CT training can improve the flexibility of students’ thinking. This process involves several related cognitive skills, including abstraction, decomposition, debugging, creativity, cooperativity, heuristic reasoning, algorithmic thinking, recursive thinking, critical thinking, problem-solving, and data analysis (Barr and Stephenson 2011; Brennan and Resnick 2012; Korkmaz et al. 2017; Wing 2006). However, not all sub-CT skills are specific and measurable. Therefore, to make the development of CT more specific and detailed, it is necessary to identify a set of CT concepts, skills, and/or practices that are specifically defined and measurable (Weintrop et al. 2016). According to the International Society for Technology in Education (ISTE 2015), there are five CT skills: namely, creativity, critical thinking, cooperativity, problem-solving, and algorithmic thinking. The Computational Thinking Scale developed by Korkmaz et al. (2017) also covers the CT skills of these five acceptable psychometric measures. CT is considered as a form of higher-order thinking; thus, CT skills are essential for every student (Grover and Pea 2019). In this study, CT skills as defined by ISTE (2015) were adopted.

CT is an indispensable part of the core skills of STEM (science, technology, engineering, and mathematics) education (Arık and Topçu 2021; Sun et al. 2020; Tan et al. 2019). They can constantly cultivate students’ ability to meet challenges in the future. How to effectively foster students’ CT skills has become a key point in educational research in recent years. Visual programming is one of the common tools for fostering K-12 students’ CT skills; it is helpful for training students’ mathematical thinking, critical thinking, creativity, and algorithmic thinking (Liu et al. 2021; Luo et al. 2020; Rodríguez-Martínez et al. 2019; Wong and Cheung 2020). Robot programming activities in STEM education are an effective teaching strategy, as they can deepen students’ comprehension of scientific concepts, improve students’ learning interest, and cultivate their creativity, critical thinking, communication, and collaboration skills (Boya-Lara et al. 2022; Jaipal-Jamani and Angeli 2017; Üçgül and Altıok 2022). However, some studies have found that visual programming learning did not have a positive influence on all higher-order thinking (e.g., Chang 2014; Falloon 2016). For example, Scratch did not affect problem solving and algorithmic thinking (Jiang and Li 2021). Thus, the way to cultivate CT skills in visual programming teaching environments should be further discussed.

Interdisciplinary approaches can foster students’ interest in learning, which can in turn cultivate their creativity and problem-solving skills (Bernstein et al. 2022). Reverse engineering pedagogy (REP), which was developed for engineering courses, involves knowledge in the field of engineering, mathematics, science, and computers. REP can instruct students to analyze existing works, deduce design parameters and implementation methods, and realize interaction between work groups (Zhong et al. 2022). The general teaching process is as follows: starting from a complete work, which can be called a “black box” (Otto and Wood 1998), students discover the design parameters and schemes of the existing work under the guidance of the teacher, and then improve or innovate the work according to the learning objectives. Different from the demonstrative method (DM), REP has the following advantages: (1) it helps students to deepen their understanding of scientific concepts and enhance their design ability in practice (Hess 2000); (2) it can improve students’ learning enthusiasm (Barr et al. 2000); and (3) it can enhance students’ learning abilities (e.g., creativity, insight, and hands-on skills) (Grantham et al. 2010; Zhong et al. 2022). Therefore, it has good applicability in robot education (West et al. 2015; Zhong et al. 2020). Some researchers have explored the effect of REP on students’ skills. For example, Ladachart et al. (2022) explored the role of REP in deepening students’ understanding of scientific concepts compared with design-based learning. Moreover, previous studies found that REP could develop students’ algorithmic thinking, problem-solving, and creativity (Abdüsselam et al. 2022; Grantham et al. 2010; Klimek et al. 2011; Tan et al. 2021). According to the definition of ISTE (2015), CT is a subset of skills including creativity, cooperativity, algorithmic thinking, critical thinking, and problem-solving. Thus, REP has the underlying ability to promote students’ CT skills. Quasi-experimental research refers to the research method that does not need to randomly arrange the subjects, but uses an original population to carry out experimental treatment under relatively natural conditions (Heath 2018). Quasi-experimental research has the basic form of experimental research, including a causal hypothesis and some types of operation that compare two (or more) conditions (Tharenou et al. 2007). Therefore, this was a quasi-experimental study which explored the effect of REP on the CT skills of primary school students in STEM visual programming robot projects.

## 2. Literature Review

### 2.1. Computational Thinking

The term CT was first proposed by Papert (1980) in his book, *Mindstorms: Children, computers, and powerful ideas.* Wing (2006) defined CT as “solving problems, designing systems, and understanding human behavior, by drawing on the concepts fundamental to computer science” (Wing 2006, p. 33). Two classification methods of CT definitions were proposed by Tang et al. (2020). The first category emphasized that CT belongs to a domain-specific field, which only covered programming and computing concepts (Denner et al. 2012; Weintrop et al. 2016; Zhang and Nouri 2019). For example, Brennan and Resnick (2012) proposed that CT included three aspects, namely, computational concepts, practices, and perspectives. The other category emphasized that CT was not limited to computer science (e.g., Guzdial 2008; Lai et al. 2021). For example, Selby and Woollard (2013) developed a CT framework including five aspects: (1) abstraction, which focuses on basic information to solve problems; (2) decomposition, which means the ability to decompose big problems into small ones; (3) algorithmic thinking, which refers to the ability to use flow charts or refine steps of problem-solving; (4) evaluation, which refers to the tendency to find the best solution to a problem; and (5) generalization, which refers to the learning transfer ability. Some researchers believe that CT is a kind of comprehensive thinking, which includes mathematical thinking, scientific thinking, and engineering thinking (Doleck et al. 2017; Korkmaz and Bai 2019). The International Society for Technology in Education (ISTE 2015) stated that CT is a problem-solving process that includes (but is not limited to) the following components: (1) data abstraction; (2) logical reasoning and data analysis; (3) the algorithm idea of automatic solution; (4) using computer-related tools to design solutions to problems; (5) efficient problem solving; and (6) learning transfer. Therefore, ISTE defined CT as comprehensive thinking and a key component of interdisciplinary teaching, which is closely related to using science, technology, and mathematical logic in hands-on operations to solve problems. Creativity, critical thinking, communication, and collaboration, which are included in CT, are seen as key skills that will help students succeed in the future (Üçgül and Altıok 2022). Therefore, this study regarded CT as a kind of higher-order thinking and explored the CT progress of primary school students.

Considering the importance of CT, CT skills should be cultivated and developed in children from an early age (Lindberg et al. 2018; Manches and Plowman 2015). STEM education provides an effective physical environment for fostering students’ higher-order thinking (Waterman et al. 2020). CT can be integrated into STEM education science courses use physical models (Arık and Topçu 2021). Visual programming learning could develop students’ CT skills (Chou 2020; Tang et al. 2020). Robot education in STEM education provides a good physical environment for visual programming, which helps to enhance students’ interest in learning; develops their creativity, critical thinking, communication, and collaboration; deepens their comprehension of scientific concepts; and improves their CT skills in practical activities (Boya-Lara et al. 2022; Jaipal-Jamani and Angeli 2017). However, some studies have found that visual programming learning did not have a positive influence on all higher-order thinking. For example, Jiang and Li (2021) discovered that Scratch programming learning did not effectively improve students’ algorithmic thinking and problem-solving ability. Problem-solving and algorithmic thinking also tend to be the weakest among students’ CT skills (Korkmaz and Bai 2019). Therefore, the influence of teaching methods on improving students’ CT skills remains to be explored in the robot visual programming environment.

### 2.2. Reverse Engineering Pedagogy and Computational Thinking

Reverse engineering (RE) originated in the field of engineering (Raja 2007). Contrary to forward engineering which emphasizes the process from ideas to projects, RE starts from a complete project, goes through a series of measurement and analysis processes to obtain a virtual model, and emphasizes understanding and overall grasping of projects (Zhong et al. 2020). The design process of engineering has been considered as a teaching method that can be used to improve students’ problem-solving and CT skills (Ehsan et al. 2021; Ladachart et al. 2022; Zhou et al. 2017). RE can be traced back to 1992 as a teaching method. Sheppard (1992) set the teaching objectives of the “Mechanical Anatomy” course to develop students’ problem-solving skills, and encouraged students to be hands-on, namely, in the “anatomy” process—disassembly and reconstruction. RE can be used in the process of developing different products based on existing components or products, namely redesign (Lee and Woo 1998).

REP conducts teaching according to certain steps. Wood et al. (2013) summarized 10-step RE and redesign approaches. The structure was divided into three phases: reverse engineering, modeling and analysis, and redesign. The first stage began with studying, forecasting, and making assumptions about the project to reduce the influence of learners’ psychological biases on learning. Then learners disassembled the project to deepen their understanding of components and projects. The second stage was the analysis and modeling. The main task was understanding the structure of projects, analyzing the existing problems, and thinking about the optimal solutions. The third stage was the redesign. Three improvement methods were proposed: namely, the parametric, adaptive, and primitive methods. According to the stages of Piaget’s cognitive development, children’s thinking develops through four stages (Piaget 1972): the sensori-motor level, the pre-operational level, the stage of concrete operations, and the formal operational stage. The development of K-12 students’ thinking involves the latter three stages. Students’ abstract thinking and logical thinking also develop from generation to maturity. Thus, teachers must set appropriate teaching objectives to guide the redesign process. Therefore, REP adopted in this study did not fully follow the 10-step reverse engineering and redesign methods proposed by Wood et al. (2013). This experiment started with the analysis and disassembly stage to learn the basics and redesign the product.

REP has formed a specific educational model during its development. Zhong et al. (2020) summarized previous studies and proposed “the Latent Model”, which included four RE instructional models, namely, (1) “Deconstruction and recovery” which means the dismantling and recovery of the project; (2) “Troubleshooting and recovery” which means solving problems in the project and restoring the structure of the project; (3) “Element minitrim”, which means deconstructing and adjusting some elements of the project; and (4) “Structural innovation” which means dismantling and rebuilding the project. Troubleshooting can effectively improve students’ ability to solve problems (Zhong and Li 2019). It is easier to tweak certain elements of a project than to innovate the structure in the teaching process. Therefore, the second and third RE instructional models were used in this experiment according to whether there were problems with the projects.

In the process of REP and the redesign approach, students can develop innovation over a “hands-on” project (Otto and Wood 1998). REP is a project-based learning strategy which could cultivate students’ communication and collaboration abilities in mechanical engineering education (Barr et al. 2000). In computer courses and engineering courses, REP could enhance students’ problem-solving skills by solving problems that arise in specific projects (Dempere 2009). REP could improve K-12 students’ creativity and self-efficacy in STEM visual programming projects (Leonard et al. 2016). REP has advantages over forward project-based pedagogy (FPP) in terms of promoting K-12 students’ creativity (Zhong et al. 2020). Concrete instructional design in STEM courses is used to improve students’ mathematical thinking, CT, and problem-solving skills (Sung and Black 2020). The International Science Education Conference 2021 (ISEC 2021) used REP to incorporate design into the physics curriculum to address the problem of unfocused goals, which greatly improved students’ problem-solving efficiency. In programming activities, it is valid to use REP to cultivate students’ logical thinking, algorithmic thinking, critical thinking, and problem-solving skills (Abdüsselam et al. 2022; Griffin et al. 2012; Rogers-Chapman 2014). Therefore, it can be inferred that REP can develop students’ CT skills in STEM learning activities, but this still needs to be confirmed in future studies. Thus, this study explored the effect of REP on CT skills of primary school students in STEM learning activities.

### 2.3. Research Question

Nowadays, interdisciplinary skills are conducive to students’ success in global competition (ISTE 2015). Thus, CT training in STEM education is very important. Existing studies have found that visual programming cannot effectively improve each dimension of higher-order thinking, such as problem-solving and algorithmic thinking, but it can effectively cultivate students’ creativity, cooperativity, and critical thinking. Previous studies (Abdüsselam et al. 2022; Barr et al. 2000; Rogers-Chapman 2014; Sung and Black 2020; Zhong et al. 2020) indicated that REP can improve students’ creativity, communication and collaboration abilities, mathematical thinking, problem-solving ability, algorithmic thinking, and critical thinking. There is less research exploring the effect of REP on primary school students’ CT skills in STEM learning activities. Thus, this research adopted a two-group pretest-posttest quasi-experimental study to explore whether REP can promote the CT skills of primary school students in STEM visual programming and robot projects. Therefore, the following question was raised.

RQ: can REP effectively improve the CT skills of primary school students in STEM learning activities?

## 3. Methodology

### 3.1. Participants

This research was launched as part of STEM learning of a primary school in Xiamen, China, from March to June 2022. The research subjects were 101 fifth graders aged 10–11 years old, who were taught by the same teacher. A quasi-experimental study was conducted. Two classes were selected as the control group (CG, 25 girls and 25 boys) and the experimental group (EG, 22 girls and 29 boys) in this primary school. Since the classes of the primary school were divided according to a random principle and students started to learn visual programming and robotics from the fifth grade, the two groups had a similar starting level in visual programming learning.

### 3.2. Learning Materials

**Figure 1:**
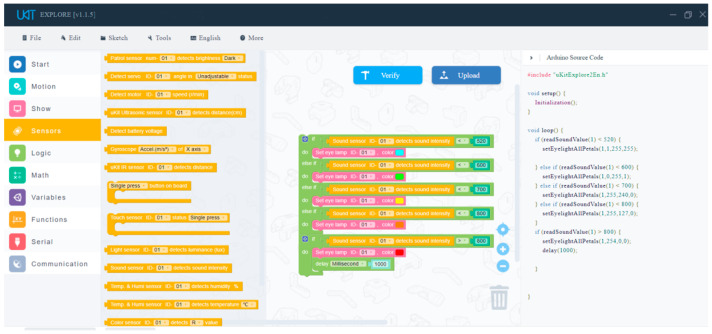
uKit Explore home page interface.

Six topics of fifth graders’ STEM learning activities related to AI were selected in this experiment: namely, Publicity Board, Noise Detector Design, Sound and Light Control Switch, Gesture Interaction, Alarm Line, and Mine. A brief introduction of each topic is shown in Appendix A. The programming platform used in this study was uKit Explore, professional visual programming software provided by UBTECH for the competition. It uses an Arduino-compatible open-source platform master controller to meet the programming needs of learners at different levels with rich learning resources. The software is compatible with the uKit servo and several structural parts specifications, and supports many programming languages. Students’ programming projects can be retained in the form of projects for communicating and displaying among students and teachers. Figure 1 shows the home page of the uKit Explore software. There are different colors in the leftmost stage which represent different functions. For example, the yellow block represents the “Sensor” function.

**Figure 2:**
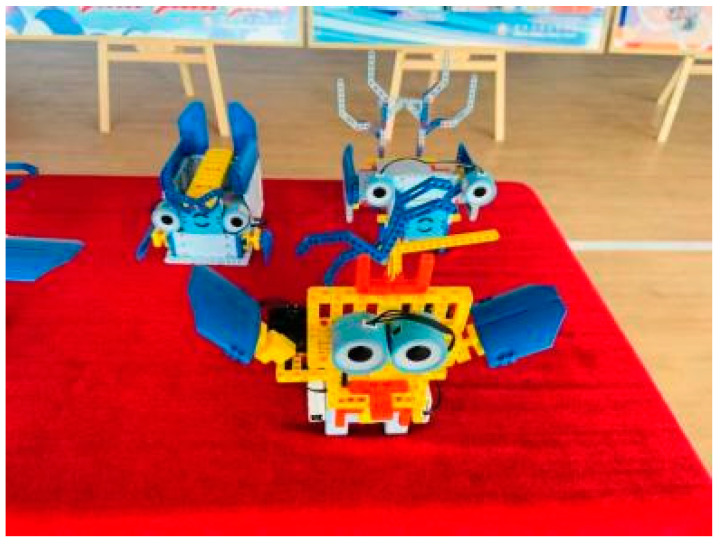
Noise detector design models.

**Figure 3:**
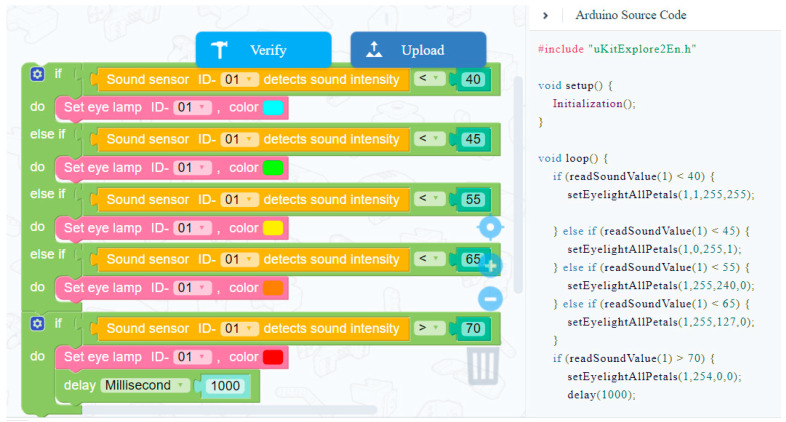
The code of noise detector design.

The general process of each project is to code and build the project according to the existing materials, carry out continuous debugging and operation, and finally finish the teaching goal. The construction of the robot project involves engineering and science knowledge, and the process of coding and debugging involves the knowledge of computer and mathematical logic, which are adapted to the content of various disciplines in STEM learning activities. One typical project was the Noise Detector Design (see Figure 2); the teaching objective of this project was that students could use the color of the tiny flashlight LED to detect the volume of the sound. To achieve this goal, sound sensors, tiny flashlight LEDs, switches, and several parts were used for construction, as shown in Table 1: Firstly, the detection department and handle were built, then the whole of the project was assembled. The project construction process involved engineering and physics knowledge, and mathematical logical thinking. Secondly, students used conditional statements (showing a blue light if the volume of the sound was less than 40 dB or showing a green light if the volume of the sound was less than 45 dB), controlling the color of the lights to indicate the volume of the sound (see Figure 3). In this process, setting code parameters and programming processes involved knowledge of computer and mathematics disciplines.

### 3.3. Procedures

**Figure 4:**
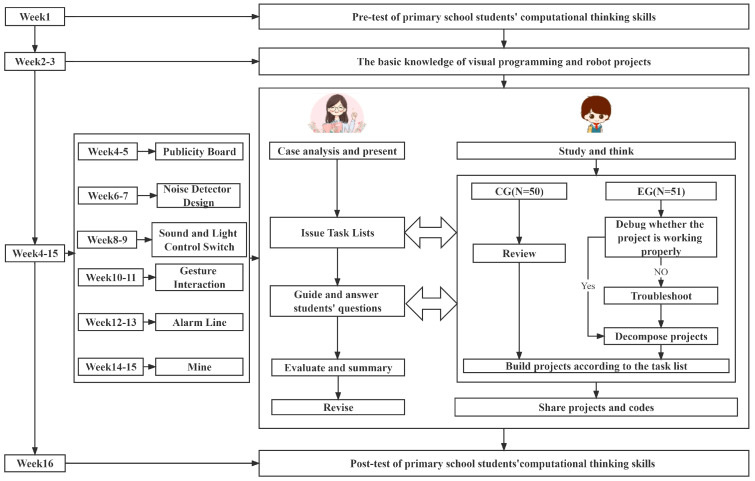
Experiment design and procedure.

The experimental process is shown in Figure 4. The same STEM course teacher taught the EG and CG, and the materials for construction and the textbooks used were the same. The teacher taught each class once a week for 16 weeks. In the pretest and posttest, questionnaires containing the CT scale were sent to the EG and CG students. Students in both groups completed the CT skills pretest and posttest. During the first week, they completed the pretest. From week 2 to week 3, the teacher taught the basic knowledge of STEM visual programming and robot projects, and taught students how to use the programming software uKit Explore. From weeks 4 to 15, the teacher assigned six visual programming and robot projects for the two classes and asked the students to complete one project every two weeks. The teacher divided each class into 10 learning groups, numbered 1–10 with five to six students in each group. In each project, the teacher’s job involved analyzing cases, providing teaching tasks, and answering questions proposed by students. The students’ learning tasks were reviewing the basic knowledge, building the project, and running it. However, the EG and CG adopted different teaching procedures. The teaching procedures of the CG were reviewing, constructing the project, demonstrating, and reporting. The teaching procedures of the EG were analyzing projects, troubleshooting and dismantling the project, rebuilding the project, demonstrating, and reporting. The CG was taught first every week. Each group in the CG constructed projects referring to the theme and experimental equipment provided by the teacher and then presented their robot projects and programming codes on the stage. Then the EG was taught, and the programming projects built by the CG were distributed to each group in the EG according to the number of the group. The teacher provided the task lists of the EG as follows: (1) debug whether the project is working properly; (2) if the programming project can run, disassemble and rebuild it according to the teaching objectives; (3) if there is a fault in the programming project, try to solve the problem, disassemble, and rebuild. After that, all the groups reported the existing problems and solutions of the original projects, the innovative points of the new projects, and the flow charts of design thinking in turn. Finally, students showed and ran the new projects, and reported the new knowledge learned. The teacher commented on the project of each group and invited representatives from other groups to make comments. In the final week, the EG and CG completed the posttest of their CT skills.

### 3.4. Instrument

ISTE (2015) stated that CT is a reflection of higher-order thinking, and divided CT skills into the following five sub-dimensions: cooperativity, creativity, algorithmic thinking, critical thinking, and problem-solving. Scholars have developed several computational thinking scales (CTSs) to evaluate the CT skills of adolescents. For example, Korkmaz et al. (2017) put forward a CTS to evaluate undergraduates in Turkish. Realizing that high school students’ CT skills in China could be better measured, Korkmaz and Bai (2019) revised the scale proposed by Korkmaz et al. (2017). The sub-dimensions of this CTS were consistent with the CT skills measured in this study. Therefore, the CTS developed by Korkmaz and Bai (2019) was translated into Chinese for this study, then adapted and simplified for the understanding and application of primary school students in China. In this study, the CTS was a 5-point Likert scale with 20 single-choice items which were divided into the following 5 sub-dimensions: (1) Creativity (3 items); an example of this subscale is: “I believe I can solve the problems that might arise when I encounter new situations”. (2) Cooperativity (4 items); an example of this subscale is: “More ideas are emerging in collaborative learning”. (3) Algorithmic thinking (4 items); an example of this subscale is: “I can immediately establish a thought process that can solve the problem”. (4) Critical thinking (4 items); an example of this subscale is: “I can use a systematic approach when comparing the options at hand and making a decision”. (5) Problem-solving (5 items); an example of this subscale is: “I can apply my planned solutions step by step”.

To further verify the applicability of this CTS to this study, 100 fifth graders from another two classes of this primary school were selected for the pilot study before the beginning of this study (McNeill et al. 2016). A total of 73 valid data were collected, and the CTS was tested for reliability and validity. The results showed that the Kaiser–Meyer–Olkin (KMO) was 0.853 > 0.800 (*p* < 0.01), the explanatory degree of cumulative variance was 70.832% > 70%, and the Cronbach’s alpha was 0.895 > 0.600. The Cronbach’s alpha of each dimension of CT skills is shown in Table 2, all of which were higher than the threshold (Alwin 1989; Alwin and Brett 2016). Although the first figure is low, the CTS could be used (Tran 2018).

### 3.5. Data Analysis

This study used the software SPSS 26.0 to analyze the data on students’ CT skills in the two classes. Firstly, the means (*M*) and standard deviations (*SD*) of the EG and CG data were calculated using descriptive statistical methods. Secondly, this study used the Kolmogorov–Smirnov Z-test to test whether the data of the two groups conformed to normal distribution. If the result conformed to normal distribution, this study used the independent samples *t* test to verify the difference in the starting and ending levels of students’ CT skills between the two classes, and used the paired samples *t* test to test the difference in the development of students’ CT skills between the two classes. If the result did not conform to normal distribution, the Mann–Whitney *U* test was performed to test the difference in the students’ CT skills’ starting and ending levels in the two classes. The Wilcoxon signed-rank test was used to verify the differences in the improvement of CT skills by students in the two classes.

## 4. Results

The aim of this research was to test the effect of REP on fifth graders’ CT skills, so the standard deviations (*SD*) and means (*M*) of each class were counted in the pretest and posttest. Cronbach’s α of the pretest and posttest were 0.850 and 0.948. If the data from each dimension of the two tests were normally distributed, this study would use the paired sample *t* test and independent sample *t* test. The results are shown in Table 3 (Dereli İman et al. 2017), and only the KS-Z of cooperativity in the pretest of EG was 0.176 > 0.05. The result did not conform to normal distribution. Therefore, non-parametric tests were used in this study.

This study used the Mann–Whitney *U* test to verify the difference in the CT skills’ starting and ending levels of students in the two classes, as shown in Table 4. The results of the pretest (creativity: U* = 0.997 > 0.05; cooperativity: U* = 0.278 > 0.05; algorithmic thinking: U* = 0.652 > 0.05; critical thinking: U* = 0.964 > 0.05; problem-solving: U* = 0.066 > 0.05) verified that there were no significant differences in the starting levels of CT skills in the two classes, which further indicated that the two selected classes were suitable for this study. The results of the posttest (creativity: U* = 0.000 < 0.05; cooperativity: U* = 0.000 < 0.05; algorithmic thinking: U* = 0.000 < 0.05; critical thinking: U* = 0.000 < 0.05; problem-solving: U* = 0.000 < 0.05) showed that there were significant differences in the ending levels of CT skills in the two classes. The results verified that the development of CT skills in the EG and CG was inconsistent.

This study used the Wilcoxon signed-rank test to compare the CT skill differences between the paired samples of the two classes, as shown in Table 5. The results showed that the five sub-dimensions of the CT skills in the two classes significantly improved, namely, creativity (CG: Z* = −5.401, *p* < 0.001; EG: Z* = −6.171, *p* < 0.001), cooperativity (CG: Z* = −5.535, *p* < 0.001; EG: Z* = −6.168, *p* < 0.001), algorithmic thinking (CG: Z* = −5.530, *p* < 0.001; EG: Z* = −6.230, *p* < 0.001), critical thinking (CG: Z* = −5.996, *p* < 0.001; EG: Z* = −6.228, *p* < 0.001), and problem-solving (CG: Z* = −5.669, *p* < 0.001; EG: Z* = −6.230, *p* < 0.001). The improvement of the EG’s CT skills was greater than that of the CG, which indicated that REP played a more positive role in developing primary school students’ CT skills than the DM did.

## 5. Discussion

CT is a key element in developing STEM learning activities (Weintrop et al. 2016; Yin et al. 2020). To cultivate K-12 students’ CT skills, the education community will continue to develop ways to develop CT skills (Waterman et al. 2020). This research used a quasi-experimental study to explore the effect of REP on CT skills of primary school students in STEM learning activities. Compared with the DM, REP is more in line with the characteristics of STEM learning activities, as it can fully mobilize knowledge in various discipline areas and can better cultivate students’ problem-solving abilities (Dempere 2009).

The research results showed that the improvement of each dimension of CT skills in the EG was significantly better than that of the CG, indicating that REP played a positive role in developing students’ CT skills in STEM learning activities, which was consistent with the previous hypothesis. Previous researchers have used diverse teaching activities in REP to explore its effect on the five sub-dimensions of CT skills. For example, in the course of mechanical engineering, REP and the learning method of group cooperation were used to construct the three-dimensional solid model, and freehand sketches and notes were used to record the mechanical decomposition process of RE during team communication, which greatly improved the students’ cooperativity (Barr et al. 2000). REP can help students solve practical problems better (Calderón 2010; Lur et al. 2022). In computer and science courses, Klimek et al. (2011) fully introduced the teaching model and strategy of creative thinking by investigating the usage scenarios of REP and listing the methods to solve practical problems, which cultivated students’ creativity and algorithmic thinking. Zhong et al. (2020) compared the different influences of FPP and REP on problem-solving and studied the Latent Model, which involved four models of REP to foster students’ problem-solving ability and creativity in a variety of teaching activities. Griffin et al. (2012) took Deconstruction Kits in REP as a tool to attract learners’ attention and to develop their critical thinking and problem-solving ability, and then improved students’ creativity through decomposition and debugging. Zhong et al. (2022) built a blue smart car produced by CFunWorld using REP. In the process of perceiving, observing, breaking down, summarizing, drawing program flow charts, restoring works, redesigning, revising and adjusting, and reflecting, students can develop hands-on skills, algorithmic thinking, critical thinking, creativity, and problem solving through cooperative learning. The experimental process of this study also followed the REP and redesign methods to develop students’ CT skills to the maximum extent. Two teaching models from the Latent Model proposed by Zhong et al. (2020) were used in this study. One concrete example in this experiment was “the Noise Detector Design project”. In the process of troubleshooting, the EG students learned the design ideas of projects and the principle of the components by analyzing or debugging the codes and the projects, while the CG students built the project using the project code directly provided by the teacher. Therefore, the problem-solving skills and critical thinking of EG students were better developed than those in the CG. In the process of disassembling and adjusting their projects, the students in the EG were asked to record the design thinking of the new project and show their ideas on stage according to the learning objectives. The students applied the design ideas of the original projects to the new ones and made innovations through learning transfer to cultivate creativity and algorithmic thinking. Therefore, when students in the EG encounter similar problems, it is easy to apply the knowledge and skills they have learned.

## 6. Conclusions and Limitations

This research was carried out in STEM visual programming and robot projects, and explored the effect of REP on primary school students’ CT skills. The results verified that REP can develop students’ CT skills better than the DM can. Specifically, REP could develop the five sub-dimensions of CT skills.

This study has theoretical and practical significance. Theoretically, REP originally belonged to the field of engineering, but the application of REP in STEM education has expanded it to interdisciplinary fields. This study verified the applicability of REP to elementary school robot education, which is consistent with the findings of Zhong et al. (2020) and Israel-Fishelson and Hershkovitz (2022). This study verified the effect of REP in primary school and provided ideas for future research on CT training methods. Teachers can consider using different teaching methods to foster students’ CT skills, which has certain reference significance for future research. In a practical sense, this study considered the effect of teaching methods, teachers, teaching time, the starting level of students’ CT skills, and other factors on the experimental results, and conducted effective control to prevent irrelevant variables from having a significant influence on the experimental results. This study applied REP to STEM teaching activities in primary schools, provided an example of the use of REP in primary school robot education, and cultivated students’ hands-on operation and learning transfer ability.

Some limitations should be considered when representing the findings. Firstly, the sample was limited to 101 fifth graders from a primary school in China. Further research can expand the sample to include other grades and regions. Secondly, the course type was limited. This study was conducted in STEM visual programming and robot projects, which mainly relied on programming and computers. Future research can extend the approach to other courses, such as unplugged projects, electronic reading, etc. Finally, the research method adopted was a quasi-experimental study, and two existing classes in a primary school were selected as the EG and CG. There was therefore no way to expand the sample size of the two groups, resulting in a small sample size. The problem of small sample size often occurs in quasi-experimental studies (e.g., Cheng et al. 2020; Cutumisu et al. 2020; Hsiao et al. 2021; Nicolaidou et al. 2021; Tang and Hew 2022; Yalçın and Erden 2021; Zhao et al. 2022). Therefore, it is necessary to expand the sample size to ensure that the experimental results are consistent with this study in future studies and to further prove the reliability of this study. Finally, this study only assessed participants’ feelings or confidence about their CT skills via the CT scale, not their actual CT skills. Future research could use different measurements to evaluate students’ CT skills.

## Figures and Tables

**Table 1:** Material bar of noise detector design.

Serial Number	Name of the Material		Quantity
1	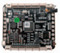	Controller	1
2	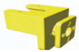	Deviator	2
3	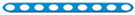	9 beams	2
4	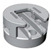	Drive coupling (wheel)	1
5	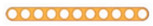	11 beams	1
6	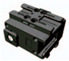	Steering engine	1
7	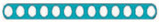	13 beams	12
8	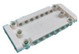	Rectangular panel (white)	4
9	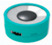	Tiny Flashlight LED	2
10	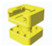	3X3 connection block with holes	2
11	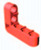	2X3 bidirectional right Angle beam	2
12	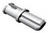	Dowel	2
13	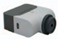	Sound transducer	1
14	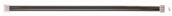	Long steering gear connection wire	2
15	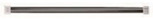	Short steering gear connection wire	1
16	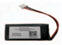	Battery	1
17	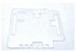	Upper acrylic sheet	1
18	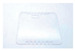	Lower acrylic sheet	1
19	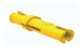	Yellow long pin	12
20	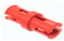	Red pin	52
21	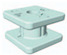	Special-shaped I-block	7
22	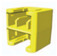	Double the square block	5
23	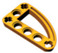	3 × 5 curved beam	2
24	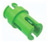	Green short pin	6

**Table 2:** Reliability of the revised CT scale.

Dimension	Cronbach’s α
Threshold	>0.7
Creativity (3)	0.698
Cooperativity (4)	0.700
Algorithmic thinking (4)	0.700
Critical thinking (4)	0.731
Problem-solving (5)	0.700

**Table 3:** Descriptive statistics of the experimental group (EG) and the control group (CG) and normality test results with Kolmogorov–Smirnov Z.

Groups	Measurements	*M*	*SD*	χ^2^	KS-Z	*p*
CG	Creativity Pretest	3.007	0.705	0.497	0.010	0.146
	Creativity Posttest	3.673	0.593	0.351	0.001	0.169
	Cooperativity Pretest	3.120	0.621	0.386	0.004	0.156
	Cooperativity Posttest	3.675	0.549	0.302	0.025	0.134
	Algorithmic thinking Pretest	3.100	0.639	0.408	0.003	0.158
	Algorithmic thinking Posttest	3.735	0.523	0.274	0.031	0.131
	Critical thinking Pretest	3.060	0.679	0.461	0.010	0.145
	Critical thinking Posttest	3.800	0.537	0.288	0.024	0.135
	Problem-solving Pretest	3.124	0.607	0.369	0.015	0.141
	Problem-solving Posttest	3.712	0.379	0.144	0.030	0.132
EG	Creativity Pretest	3.020	0.707	0.500	0.005	0.152
	Creativity Posttest	4.516	0.661	0.437	0.000	0.258
	Cooperativity Pretest	3.020	0.581	0.337	0.176	0.110
	Cooperativity Posttest	4.451	0.640	0.410	0.000	0.209
	Algorithmic thinking Pretest	3.201	0.623	0.388	0.000	0.195
	Algorithmic thinking Posttest	4.539	0.673	0.453	0.000	0.250
	Critical thinking Pretest	2.918	0.465	0.216	0.002	0.160
	Critical thinking Posttest	4.500	0.665	0.442	0.000	0.284
	Problem-solving Pretest	2.918	0.456	0.216	0.003	0.159
	Problem-solving Posttest	4.643	0.424	0.180	0.000	0.231

Note: EG = the experimental group; CG = the control group.

**Table 4:** Mann–Whitney *U* test results from the pretest and posttest scores of the experimental and control groups.

	Group	*N*	Mean Rank		Sum of Rank		*z*		U*	
			Pretest	Posttest	Pretest	Posttest	Pretest	Posttest	Pretest	Posttest
Creativity (3)	CG	50	51.01	34.24	2550.5	1712.0	−0.003	−5.786	0.997	0.000
	EG	51	50.99	67.43	2600.5	3439.0				
Cooperativity (4)	CG	50	54.16	34.51	2708.0	1725.5	−1.085	−5.650	0.278	0.000
	EG	51	47.90	67.17	2443.0	3425.5				
Algorithmic thinking (4)	CG	50	49.69	33.99	2484.5	1699.5	−0.452	−5.849	0.652	0.000
	EG	51	52.28	67.68	2666.5	3451.5				
Critical thinking (4)	CG	50	50.87	35.18	2543.5	1759.0	−0.045	−7.574	0.964	0.000
	EG	51	51.13	66.51	2331.0	3392.0				
Problem-solving (5)	CG	50	56.34	28.89	2817.0	1444.5	−1.837	−7.574	0.066	0.000
	EG	51	45.76	72.68	2334.0	3706.5				

Note: EG = the experimental group; CG = the control group; * Statistical significance level has been adopted as .05/5 = .01 for this analysis using Bonferroni correction.

**Table 5:** **The** Wilcoxon signed-rank test on the CT skills’ five sub-dimensions of the two classes.

	Group	*N*	Mean Rank	Sum of Ranks	Z*	*p*
Creativity (3)	CG	50	19.500	741.000	−5.401	0.000
	EG	51	25.500	1275.000	−6.171	0.000
Cooperativity (4)	CG	50	20.500	820.000	−5.535	0.000
	EG	51	25.500	1275.000	−6.168	0.000
Algorithmic thinking (4)	CG	50	20.500	820.000	−5.530	0.000
	EG	51	26.000	1326.000	−6.230	0.000
Critical thinking (4)	CG	50	24.000	1128.000	−5.996	0.000
	EG	51	26.000	1326.000	−6.228	0.000
Problem-solving (5)	CG	50	21.500	903.000	−5.669	0.000
	EG	51	26.000	1326.000	−6.230	0.000

Note: EG = the experimental group; CG = the control group; * Statistical significance level has been adopted as .05/5 = .01 for this analysis using Bonferroni correction.

## Data Availability

The original contributions presented in the study are included in the article/Appendix A, further inquiries can be directed to the corresponding author.

## Appendix A

**Table A1:** Information on the control group’s six items.

Weeks	Project’ Name	Teaching Objectives	Works and Codes
4–5	Publicity Board	Learning objectives: (1) Students learn to use related components, brightness sensors, and tiny flashlight LEDs. (2) Students understand the meaning and usage of the function blocks: “if…… so…” and “Otherwise”.	
		Learning content: (1) Students build a propaganda window, propaganda support frame, and operation platform. (2) Students perform visual programming to realize the function that the light changes with the intensity of light outside.	
6–7	Noise Detector Design	Learning objectives: (1) Students learn to use related components—sound sensors and tiny flashlight LEDs. (2) Students understand the meaning and use methods of the function block: “if…… perform… otherwise if…… perform…”.	
		Learning content: (1) Students build the test section, handle (handheld part), and assemble the whole project. (2) Students perform visual programming to light the color of the lamp flap according to the volume of the sound.	
8–9	Sound and Light Control Switch	Learning objectives: (1) Students learn to use relevant components, sound sensors, and brightness sensors. (2) Students understand the meaning and usage of the logic function block: “And”.	
		Learning content: (1) Students build the first layer including the fixed brightness sensor, sound sensor, and tiny flashlight LEDs, and place the motherboard and battery in the second layer. (2) Students perform visual programming to realize that the light will be on for 10 s when the light outside is dimmed or there is sound.	
10–11	Gesture Interaction	Learning objectives: (1) Students learn to use related components, infrared ranging sensors. (2) Students understand the meaning and usage of modules: “Repeat…… Perform…”. (3) Students learn to modify ID.	
		Learning content: (1) Students build the testing department, operation table, and overall assembly. (2) Students perform visual programming to realize the function: “swing from left to right”.	
12–13	Alarm Line	Learning objectives: (1) Students learn to use related components, infrared ranging sensors. (2) Students understand the logical function block: “if…… so…”.	
		Learning content: (1) Students build a base, left and right-side panels, back plate, cover plate, and front, and assemble the project. (2) Students should carry out visual programming to realize the function of the warning line to pass or obstruct by identifying car models.	
14–15	Mine	Learning objectives: (1) Students understand the use and setting methods of the “sound effect module” and “light module.”	
		Learning content: (1) Students build the upper layer and the lower layer and assemble the project. (2) Students carry out visual programming to realize the function of simulating an explosion when the switch is pressed and the buzzer sounds.	

**Table A2:** Information on the experimental group’s six items.

Weeks	Project’ Name	Teaching Objectives	Works and Codes
4–5	Publicity Board	Learning objectives: (1) Students learn to identify problems in analysis. (2) Students learn the basic knowledge of the project in the process of dismantling it.	
		Learning content: (1) Students analyze whether the project can run normally. (2) Students troubleshoot if there is a fault. (3) Students disassemble the project, change the appearance of the project to make it more concise and beautiful, change the standard of lighting change with the light intensity, and explain the reasons for setting this standard.	
6–7	Noise Detector Design	Learning objectives: (1) Students learn to identify problems in analysis. (2) Students learn the basic knowledge of the project in the process of dismantling the project.	
		Learning content: (1) Students analyze whether the project can run normally, and troubleshoot if there is a fault. (2) Students disassemble the project, change the appearance of the project to make it more creative, change the standard of sound and light colors, and explain the reasons for setting such standards.	
8–9	Sound and Light Control Switch	Learning objectives: (1) Students learn to identify problems in analysis. (2) Students learn the basic knowledge of the project in the process of dismantling it.	
		Learning content: (1) Students analyze whether the project can run normally. (2) Students troubleshoot if there is a fault. (3) Students disassemble the project, change the appearance of the project to make it more concise, change the standard of lighting changing with light intensity or sound, and explain the reasons for setting this standard and whether the intensity of light is related to seasonal changing.	
10–11	Gesture Interaction	Learning objectives: (1) Students learn to identify problems in analysis. (2) Students learn the basic knowledge of the project in the process of dismantling it.	
		Learning content: (1) Students analyze whether the project can run normally. (2) Students troubleshoot if there is a fault. (3) Students disassemble the project, change the appearance of the project to make it more creative, change the direction of the gesture changing, and realize the swing “from right to left”.	
12–13	Alarm Line	Learning objectives: (1) Students learn to identify problems in analysis. (2) Students learn the basic knowledge of the project in the process of dismantling it.	
		Learning content: (1) Students analyze whether the project can run normally. (2) Students troubleshoot if there is a fault. (3) Students disassemble the project and add models that can be identified to achieve faster release.	
14–15	Mine	Learning objectives: (1) Students learn to identify problems in analysis. (2) Students learn the basic knowledge of the project in the process of dismantling it.	
		Learning content: (1) Students analyze whether the project can run normally. (2) Students troubleshoot if there is a fault. (3) Students disassemble the project, change the appearance of the project to make it more creative, change the standard of pressure, and change the color of the lights and the explosive music.	
